# How educational systems respond to diversity, inclusion and social justice: Disability, power, discipline, territoriality and deterritorialization

**DOI:** 10.1111/1468-4446.12969

**Published:** 2022-07-17

**Authors:** Navin Kikabhai

**Affiliations:** ^1^ School of Education University of Bristol Bristol UK

**Keywords:** deterritorialization, disability, diversity, framing, inclusion, inclusive education, politics, representation, social justice, territorial boundaries, widening participation

## Abstract

This paper presents a critical examination of a vexed issue relating to how educational systems respond to diversity, inclusion, and social justice. Whilst there are unique factors specific to the various educational sectors; that is, to early years, schools, colleges, higher education and to the life‐long learning sector, this paper explores education and diversity in its broadest sense and recognizes that issues are as much cross‐sector as they are within‐sector. Further still, this paper shifts across disciplinary epistemic boundaries making use of Foucault's tools and the work of Deleuze and Guattari. Given this broader context, this paper primarily traverses the borders of schooling and higher education. It utilizes the notion of scales of justice and draws upon the work of Fraser and explores how this can offer insights into issues not only in relation to redistribution and recognition, but also to representation. It intentionally, draws upon (critical) disability studies literature; and the often‐forgotten discrimination known as disability. It acknowledges the various paradigms and terminological descriptors associated with disabled people, how these are intentionally, I argue, produced and re‐produced, subject to a process of misframing, misrecognition and maldistribution through various territorialized and often segregated educational spaces. In response, this paper offers a reading of dis/ability which moves through theoretical and conceptual understandings and advances the notion of deterritorialization in order to escape, engage and identify larger patterns of inequality. It offers different insights, provides an alternative mapping that can raise different critical questions about disability, also to issues of diversity, inclusion, and social justice.

## 1 INTRODUCTION

Whilst there are unique factors specific to the various educational sectors; that is, to early years, schools, further education colleges, higher education and to the life‐long learning sector, this paper explores education and diversity in its broadest sense and recognizes that issues are as much cross‐sector as they are within‐sector (Ainscow et al., 2006; Barton & Slee, 1999; Wright et al., 2006) and in some instances this also extends to the workplace (Parry, 2010). Afterall, as Peters (2010, p. 149) noted from a global perspective, disabled people face injustice and discrimination “*regardless of national boundaries*”; whether these are global or within education itself. Certainly, ending barriers experienced by disabled people is as much about making transparent territorialized boundaries between sectors as well as within sectors and across disciplinary fields. Indeed, as Paasi (1999, p. 84) noted boundaries are “*one part of the ‘discursive landscape’ of social power, which is decisive in social control and the maintenance of social order*”. As is noted by Vollard (2009) territory matters, and that this also involves:

… scrutiny of political institutions, social practices, representations, ideologies, images, discourses and symbols by which territorial demarcations are established, consolidated, and maintained. (Vollard, 2009, p. 28)

For sure, surveying territories of knowledge(s) which often shape understandings of equality, social justice and by association notions of diversity, and social mobility; also matters. In unison to this and within the context of higher education this includes notions of widening participation. For example, Reay (2017), with respect to social class and borrowing from Stephen Ball, made this point:

There is only so much that educational institutions can do to improve social class inequalities, given the economic and social context in which they operate. As Ball, concludes, schools are increasingly the wrong place to look if we want to do something about class inequalities in education. (Reay, 2017, p. 43)

For clarity, Ball (2010, p. 164) suggests looking at the commodification of the “social” through a plethora of educational services fueled by lucrative (aggressive) market forces which instil fear and insecurity in parents' sensibilities, caught across boundaries, focusing on “*relational advantage*” and little if any “*consideration to the fate of others*”. Of course, this middle‐class “*relational advantage*” is out of reach for many (disabled and non‐disabled) parents of disabled children (Wilde & Hoskinson‐Clark, 2014), even though there are policy claims giving such parents personal budgets and so‐called “choice” (D*f*E & D*f*H, 2015). It may also be equally useful to look elsewhere.

In another domain, UK higher education has been marked by an agenda of widening participation and has primarily been monitored in relation to socio‐economic status, age, gender, religion, ethnicity, sexuality, and less so disability (Deem & Morley, 2006; Kikabhai, 2018). Interestingly, it took a second edition of “*Fractured Identities*” (Bradley, 2016, p. 8), first published in 1996, to acknowledge disability; albeit only a “*briefer look*”. Whilst such social groups have generally increased their higher education participation rates, these have tended to be measured on single identity characteristics. Such approaches to recognition involve, arguably, what Fraser (1997, p. 25) notes as being a “*practical recognition effect*”, a stigmatizing result of its “*official recognition comment*”. As will become apparent, and argued elsewhere (Titchkosky & Mickalko, 2009, p. 5), the study of disability is a “*commitment to normalcy*”. Moreover, in terms of access to higher education, focus has generally been on individuals having to reach what is considered the “gold standard” [a so‐called measure of student success an “*illusion of meritocratic opportunity*” (Payne, 2018, p. 24)], a so‐called “*royal road*” (David, 2010, p. 11), and access through attaining A‐level results. According to Lawler (2008, p. 135) who discussing working‐class identity, suggests that the discourse of widening participation, associated social policy; is one of “*lack*”, a consistent deficit perspective. Arguably, this discourse is operationalized as a territorialized modern dualistic narrative; lack as opposed to plenty (lack/plenty). This associated policy context is laden with ableist and disablist rhetoric—coercive, perpetuating and privileging notions of ableism, normalcy, the “*ideal, normal and the mean or default*” (Dolmage, 2017, p. 7). Indeed, for Davis (1995, p. 2), “*Normalcy and disability are part of the same system*”. Even more, there has, through various higher education access initiatives such as: foundation degrees, outreach open‐days, events and programmes, been a growing focus on areas of first generation, low‐income, women, and specific ethnic groups. Relatively little attention has been paid to mature students, part‐time students, students without A‐levels, disabled students, students having completed Access courses, and also to a lesser extent that which questions curricular and assessment procedures. Further still, whilst Deem and Morley (2006) identified a wider policy context under the then UK New Labor government and features of the different forms of inequality, little attention has been directed at how higher education institutions are duty bound to change, and requirements under legislative requirements (Equality Act, 2010) to respond to diversity and make reasonable adjustments. As Bradley (2016) notes the now Equality and Human Rights Commission, a single equalities body, has replaced previous equality commissions.

Across other borders there are those who discuss campus climate and advance a model for “diverse learning environments”; link micro (students) and macro (the institution) level contexts that are themselves subject to sociohistorical forces (Hurtado et al., 2012). Others who explore academic ableism in relation to disability and higher education; offering an affirmative perspective (Dolmage, 2017), others who offer a “student experience practitioner model” (Morgan, 2013), yet still others who advance a culturally responsive pedagogy (Pirbhai‐Illich et al., 2017).

As such, this paper examines misframing of modern higher education participation with reference to questions about its purpose and function, social mobility, the politics of disability, the politics of higher education participation and how educational systems respond to diversity, inclusion and social justice. Connections are made with recognizing the dynamics of a social system, mechanisms of social selection, and institutional violence using a Foucauldian/Deleuze‐Guattarian analysis. It also highlights the use of “able” bodied metaphors as an attempt to advance social theory; yet results, as will be noted, in disability erasure. It identifies discussions which shape changing terminological descriptors across territorialized spaces, raising concerns about participatory parity. It links to educational policy, legislative changes, and identifies teacher education and postgraduate courses that reveal and perpetuate a disablist cultural capital; understandings which also perpetuate multiple inequalities. Finally, this paper interlinks issues of power, discipline, territoriality and deterritorialization using the work of Deleuze and Guattari to highlight boundaries of segregated provision. It deconstructs the territorial dis/ability divide, identifying dominant and dominating paradigms, it identifies complex social orderings, and addresses issues of diversity, inclusion, and social justice.

## 2 ADDRESSING ISSUES OF SOCIAL JUSTICE—RECOGNITION, REDISTRIBUTION AND REPRESENTATION

In brief, Fraser (1997) offers a critique of a postsocialist condition in relation to three features. First, a vision of social transformation for a just social order. Second, a shift in the grammar of social justice; and third, economic liberalism. Fraser scrutinizes the distinction between culture and the economy by decentering recognition and redistribution in terms of growing gross inequality and aggressive marketization. Fraser integrates both recognition and redistribution arguing that neither can remedy injustice alone. In a political exchange, Fraser (2003) offers a ‘perspectival dualist’ analysis and argues that recognition and distribution are irreducible. Later, Fraser (2008) explores justice in relation to modern territorial states, critiquing the Westphalian frame, and offers a third dimension, principally, representation. In short, Fraser advances the previous two‐dimensional understanding of justice and offers a three‐dimensional theory of justice. For Fraser participatory parity evokes the political and enables the identification of misframing.

## 3 MISFRAMING MODERN HIGHER EDUCATION PARTICIPATION

Whilst there is a plethora of models related to improvement, development, quality assurance, and teaching strategies/approaches promoting educational participation, those that have related to disability have tended to be conceptually framed (arguably misframed) by an individual deficit medical model of disability,^1^ indeed there have been opponents whose work has been to repeatedly dispute such thinking and practice (Oliver, 1990, 1996; Oliver & Barnes, 1998, 2012), and from which each is concerned with the “… *ordinary‐political representation to the principle of participatory parity*” (Fraser, 2008, p. 70). Interestingly, this principle is encapsulated in the title of the edited work by Swain and French (2008) “*Disability on Equal Terms*” which seeks to confront injustices (disablism) experienced by disabled people and simultaneously advances an affirmative model aligned with a broader disability arts movement and an assertion of identity politics. Although, as argued by Fraser (1997, p. 29); affirmative recognition could possibly fuel “*flames of resentment*”. Elsewhere Roulstone and Barnes (2005) have been addressing issues related to the political economy, suggesting that the:

… terrain [territory] of discussion should widen beyond simply that of access to paid work and the need to look more creatively at the economic and social contribution made by disabled people outside of paid work, … (Roulstone & Barnes, 2005, p. vii, my insertion)

Arguably, the authors are, in Fraser's (1997) terms, deconstructing what it means to “work”. Anitha and Pearson (2018), who document the experiences of striking South Asian women, too are suggesting restructuring industrial relations in favor of Fraser's (1997) analytical framework which integrates recognition and redistribution. For Fraser (1997, 2003; 2008) exploring injustices of recognition and redistribution have advanced political‐philosophical debate into reconciling the redistribution‐recognition dilemma. In the context of higher education, pertinent questions are being asked in terms of its purpose, its function, who they include and exclude, principally, in this case, concerning the politics of disability, the politics of higher education participation (Kikabhai, 2018), and of representation. More broadly, a searching question was asked in an edited publication, published in the first designated International Year of Disabled People, asking “*Is Higher Education Fair?*” (Piper, 1981). In a contributing chapter Williamson, using a social class analysis, noted that:

… where social mobility depends more than ever on education certification. White collar workers, technical workers, professionals and semi‐professionals are the groups which have benefited most from the expansion of higher education. Is it realistic to expect that they will settle for something less in the future? I ask this without knowing the answer but suspecting that whatever happens to higher education in the coming few years, those who have historically had little access to it will be those less likely to be consulted about how it should change or to benefit from the changes which will take place. (Williamson, 1981, p. 38)

In Weber's (Gerth & Mills, 1974) terms exploring this institutional violence involves an examination of rationality, the (bureaucratic) apparatus and distribution of power, and in Marx's terms this involves exploring the experience of alienation (Lőwith, 1993); alongside, material relations, commodification, and capitalist modes of (academic) production. This, no doubt, raises a question of representation, and if, indeed, whether higher education has a future at all (de Sousa Santos, 2017), and it seems this is irrespective of whether it is considered as being fair. Rather, given a possible post‐Covid‐19 future, the university machine has jammed, and instead is scrambling for new online and virtual territorialized spaces. For sure, Dolmage (2017, p. 122), in the context of recognizing and acknowledging the injustice against disabled people, cautions against retrofit approaches and in the context of this impeding restructuring suggests that, “*Change … is framed as deformation, and a transgression of not only space but time*”, arguing that the retrofit (an afterthought rather than being central to the restructuring process) is typically “additional” to or to “fix” later in some way.

Certainly, within the US context, the question relating to the purpose of higher education has been earlier addressed by Ryan and Sackrey (1984, p. 257, 282) who collated essays of personal reflections and perspectives of working‐class academic staff who discuss working within an “*insane system*” and relate to notions of “*escape*”. Moreover, the authors maintained that whilst there is a definite allure to higher education participation one of its primary functions is to mask the myth of upward social mobility, noted to be “*a grand lie*” (*ibid*, p. 317), and not just for students (Crozier et al., 2010). Incidentally, hooks (1994, p. 4), also made the point that the academy is not paradise and likened it to “*a prison, a place of punishment and confinement rather than a place of promise and possibility*”. Dare it to be stated, a sobering thought, that when the Khmer Rouge took political control of Cambodia, they converted schools into prisons (Collins, 2008); no doubt their similarities are uncanny (Foucault, 1977).

Similarly, exploring the experiences of working‐class students in “elite” universities, Reay et al. (2009, p. 1108, 1110) also explored the issue of higher education participation and inquired as to whether such students are likewise *strangers in paradise*, they reported that their experiences tended to be marked by chance events, a “*clueless serendipity*”, their trajectories were facilitated by often the “*support* *of one individual teacher*”, and although none of the participants thought they were strangers, they did think that higher education participation was not paradise. Critically, and admittedly at least for Ryan and Sackrey (1984), neither of the studies involved the representation of ethnically diverse participants nor disabled people as part of their investigations. Although, arguably, there is a common thread, for example, when Ryan and Sackrey (1984) ask whether participants would repeat the choices they made, specifically in relation to working within the academy, the majority said ‘no’, which resonated with Amy's response as a student in Reay et al. (2009, p. 1111) study who disclosed that “… *I wouldn't recommend it to anyone else, particularly*’. A further interest in Reay's (2017) study is reference to Young (2002, p. 43), whose satirical sociological essay, concerning meritocracy, reflects back from the year 2034 and wrote “*The educational ladder was also a social ladder*” as well as morphing from the “*bottom*” requires a “*new accent*”. Correspondingly, Warikoo (2016, p. 7, 164) discovered in her project which also relates to the conception of meritocracy and of “race” found that not only does accent mean a lot but that within the British context “elite” higher education is “*complicit in reproducing that inequality, in part through admissions systems*”, although reading comments from students at Oxford “*there is little speaking up*” about racism, class bias or crossing any other “*hurtline*”; apparently such discriminations are passed‐off as jokes. The idea that social mobility involves working‐class students acquiring a new accent is also found across educational boundaries. For example, Crozier et al. (2010, p. 71) focusing on access, participation and diversity questions in relation to post‐compulsory further and higher education noted that working‐class students found themselves being “*forced in acts of reinvention*” and there was “*evidence of the need to change an accent …*”.

More broadly, David (2010, pp. 4–5) with additional contributing authors, noted that whilst there has been an increase in participation in higher education in the twenty‐first century “*policies have not led to fair or equal access to equal types of higher education* …”. Moreover, whilst Reay (2017) further still explored the miseducation of the working classes, Ryan and Sackrey (1984, p. 105) much earlier argued that “*The promise of class mobility underlies the ideological justification for inequalities within capitalism*” and alternatively offers the “… *basic characteristics of the dynamics of the social system and the place of the academy in the order of things*”.

Put succinctly the “order of things” not only the “order of discourse” (Foucault, 1981) is primarily about how the academy is part of the problem, a contradiction of democracy and is an arbiter that produces and reproduces inequality, competition, and so‐called academic rank, points that are ignored by Reay et al. (2009), Reay (2017) and others (Sperlinger et al., 2018). Moreover, Reay et al. (2009) also raise the issue of both belonging and escape, in this instance in relation to working‐class connections, and yet, arguably, in contrast there is also merit in “escape” from modern higher education institutions, given that they have “*transformed, consisting of wheels within wheels, a slow moving juggernaut, and increasingly subject to market forces*” (Kikabhai, 2018, p. 240). Arguably, in Foucauldian (1981) terms, the issue associated with the ideological justification of social mobility is a controlled discourse, encapsulated in power/knowledge, that has long before been spoken. This omission (Reay et al., 2009; Sperlinger et al., 2018) is arguably a kind of Mercator projection, a distortion of territorialized spaces, actions, and violent paradigmatic effects, invariably serving to exclude the already estranged. Nonetheless, Reay (2017, p. 178) does later point at “elite” universities which are the “*primary engines*” with elitist processes that masquerade as meritocracy that operationalize a “*mechanism of social selection*”, although had earlier linked this to the economy (Reay, 2013, p. 668). For sure, social mobility is a “*mirage … a figment of imagination brought to life in policy and political rhetoric*” (Reay, 2013, p. 662), an “*optimistic fantasy*” (Reay, 2018, p. 146), and nor is this about “re‐making” or resetting the university, as some would have it (Sperlinger et al., 2018), but fundamentally about the politics of higher education participation and questions of redistribution, recognition and representation. Arguably, discussions about “escape” attempts; being uni‐directional rather than bi‐ or multi‐directional, participation in higher education and (one‐generational “upward”) social mobility are as much about modern culture, recognition and an individual's search for “*meaning, novelty and their own true identity*” (Cohen & Taylor, 1992, p. i) and how the individual is constructed.

Relatedly, in *Decolonizing the University*, de Sousa Santos (2017, p. xi, xiii) makes clear that the past 4 decades, “… *the university has been undergoing a process of paradigmatic change* …” and has become an “*additional problem*”. In decolonization terms, it is not the question “*how can the university address the role it has played in reproducing global inequalities?*” (Icaza & Vázquez, 2018, p. 115) but rather “how can the university address the role it has played and continues to play in reproducing global inequalities?” that should be asked. Moreover, Kikabhai (2018) with respect to disability and the rhetoric of widening participation and in conjunction with those labeled as having “learning difficulties” (a fictious entity^2^) are:

… beset by multiple barriers; the impaired mind/body has become a problem, territorialized and subject to control and (mis)treatment. (Kikabhai, 2018, p. 240)

Definitions of disability being medical, biological, psychological, legal, educational, pseudo‐scientific and others abound; Foucault's (1977, p. 11) “*army of technicians*” for sure. Such definitions (or frames) embody a different kind of territorialized and segregated world, in which students conform to educational norms in a coercive and aggressive higher education institutional culture.

## 4 LEGISLATIVE CHANGE, “ABLE” BODIED METAPHORS, AND ERASING DISABILITY

So be it, of particular importance within the literature related to disability and higher education since the 1990s are the legislative changes that have affected the education and employment rights of disabled people. For example, the Disability Discrimination Act (1995), the Special Educational Needs and Disability Act (2001), UK government recognition of British Sign Language as a language in 2003, the Disability Equality Duty (2006), and the Equality Act (2010) requiring public bodies [*sic*] to actively promote equality for disabled people have all contributed to this changing reterritorialized educational landscape. Relatedly, Ahmed's (2012, p. 26) work considers how diversity can become an “*institutional end*”, and uses the idea of the higher education institution being understood as a “body” (or a machine), as a metaphor, specifically:

The institution, in being imagined as an organic body, is understood as a singular entity made of multiple interrelated parts, all of which contribute to the health or well‐being of that body. Indeed, organic and mechanical metaphors are used simultaneously as ways of describing the institution. Both metaphors work to convey an entity that is made up of parts, where the communication between parts is essential to an overall performance. (Ahmed, 2012, pp. 28–29)

Ahmed later goes on to argue that “*institutional racism becomes part of the institutional language*”, and that the institution becomes the “*sick person who can be helped by receiving the appropriate treatment*” (Ahmed, 2012, p. 47). Without doubt, so‐called normalcy and notions of ableism are being advanced (Titchkosky & Michalko, 2009). On the contrary, and far from this metaphor working, a kind of tree logic (Deleuze & Guattari, 2008), there are many so called “sick people” who are not racist, and certainly not disablist (Begum, 1992; Konur, 2004; McDonald, 1996; Vernon & Swain, 2002). Arguably, disabled people are made invisible, disability, that is its discrimination, is being erased. Unsurprisingly then, that almost 3 decades earlier feminist research specifically focusing on social justice, identifying “*gaps and silences*” and its calling to account for the “*complexity of all forces of identify formation*” (Arnot & Weiler, 1993, p. 213) continues to be ignored. Incidentally, it is also ignored elsewhere (UNICEF, 2018).

## 5 TERRITORIALIZED EDUCATIONAL SPACES, MORE DISABILITY ERASURE AND DISABLIST CULTURAL CAPITAL

In critically examining the changing terminological descriptors in which the lives of disabled people are shaped and reshaped across the various territorialized spaces and educational sectors, it is worthwhile stating that such labeling is not natural nor neutral. Indeed, as Slee noted with reference to schooling and a growing “special” education industry:

Special educational needs became an all‐embracing metaphor for the defective child. In turn, the functional label of ‘special educational needs’ has metaphorically become a refugee camp for the casualties of schooling. (Slee, 1998, p. 102)

For Slee (1998, p. 103) this is part of an “*enduring episteme*”. As individuals attend compulsory schooling there is a complex pathologizing bureaucratic machine of (mis)framing and labeling, operating “… *by the determination of degrees of deviance* …” (Deleuze & Guattari, 2008, p. 197). For example, disabled young people and those labeled as having “special educational needs” (SEN) are assumed to be one and the same, when there are disabled young people who do not have labels of SEN and that there are individuals said to have SEN who are not disabled young people, although they are often subject to the same discriminations (Corbett, 1996; Haines & Ruebain, 2011). By inference, deconstructing this territorial dis/ability divide, also, arguably, acknowledges that there are individuals without impairments who are disabled, that is, there are disabled people without impairments. For those labeled as having “SEN”, previously said to be approximately 20% of the schooling population in the UK of which 18% were identified as being part of the mainstream (Warnock, 1978), and that between 2% and 3%, a later conceded point that this was “*a kind of guess*” (Warnock, 1996, p. 54), who are Statemented and accompanied (or at least should be) by an Educational, Health and Care plan (D*f*E & D*f*H, 2015), are said to require “special education provision” often in the form of a segregated “special” or “specialist” school. Moreover, Warnock in an about turn, publicly described the Statementing process as a “*real evil*”, and later added that it “*is wasteful and bureaucratic, and causes bad blood*” (Warnock, 2005, p. 54) between parents and authorities. Apart from disabled people themselves being denied participatory parity as active members on the Warnock inquiry panel, this was also no doubt fueled by the rise of the so‐called “professional”, in an “*age of disabling professions*”, when people have “problems” and so‐called “*experts had ‘solutions’*” whilst “*scientists measured imponderables such as ‘abilities’ and ‘needs’*” (Illich, 1977, p. 11). For sure, trainee teachers and university students on postgraduate courses can have “special” programmes and “special” education units staffed by “special” educators where they are “*taught about human defect*” (Slee, 1998, p. 106; Slee, 2018) who in turn become tomorrow's tutors and trainers, caught‐up, implicated and entangled in a different modern performative regulatory bureaucratic machine with little chance of escape, some converting and cashing‐in on their disablist cultural capital, a “*site of re‐affirmation*” presented under the guise of rationality (Schick, 2010, p. 76) rather than challenging institutional infrastructures, segregated territorialized boundaries, and educational/epistemic violence/injustice (de Sousa Santos, 2008; Fanon, 2004). Incidentally, associated “special” education essays are an open invitation to prejudice where everything becomes a disorder, disease, disadvantage, dis … abnormality … dis … syndrome … dis … suffering … dis … defect … dis … special … dis … challenging … dis … psy … tragedy; fixated as they are on individual impairments, “types” of SEN, deficit and disablist ideologies; and even more so when it comes to notions of so‐called “learning difficulties” (Buchner et al., 2021). One can reflect of course on this intentional turn of events, and wonder: Why are educational practitioners fixated on medical understandings of disability, ought they not be studying medicine instead? Normalcy, so much for the “*good life and the only life worth living*” (Titchkosky & Michalko, 2009, p. 7); although it may be more appropriate to reinvoke the title from Klee et al. (1988) earlier work “*The Good Old Days*” to describe this intentional state of affairs; at least in a territorialized modern form. Borrowing from Fanon (2004), in this territorialized educational space where the wretched of the Earth exist, profiteers, training and shaping colonized minds, perpetuating multiple inequalities, exerting its epistemic violence for future reproducibility, where inequality, segregation, and exclusion remain the order of things, rather than pursuing epistemic justice and embracing culturally responsive pedagogy (Pirbhai‐Illich et al., 2017), creating accessible and enabling spaces, understanding the cultural and political struggle of excluded groups, human diversity, inclusion and social justice. Instead, this, modern higher education institution, a slow‐moving juggernaut, bureaucratic, engaging in a rhetoric of widening participation, being a form of violence in which “*all are equally powerless*” a form of “*tyranny without a tyrant*” (Arendt, 1970, p. 81), gives rise to new modes of surveillance, control, regulation, discipline, punishment and exclusion (Kikabhai, 2018). As is noted educational difficulties/discriminations are “*deeply entrenched*” (Ainscow et al., 2006, p. 17) within English educational policy and practice and its dominant frame is not only one of lack but also of individual deficit.

## 6 POWER, DISCIPLINE, TERRITORIALITY AND DETERRITORIALIZATION

Given the (mis)framing of SEN and disability it should be no surprise that debates about the place of disabled young people within mainstream or “special” provision are circular, and as argued elsewhere, has an intentionally strong resistance to closure (Kikabhai, 2018). Indeed, inherent in this contentious debate is in Nietzschean terms the master/slave dialectic, the deficit and exclusionary language which authors uncritically adopt and from which there is seldom any escape. With respect to the schooling sector, such provision has been restructured under a plethora of alternative, and nonetheless segregated, provision under a terminological and shifting (de)territorial boundary with academies, “free” schools, academy “specialist” schools, alternative provision, pupil referral units to name but a few.

Whilst the discipline of International Relations has been discussing the “*end of boundaries*” (Albert, 1999, p. 62) and a geography of education is “*awakening*” (Thiem, 2009, p. 155) within this educational context, new boundaries of segregated provision have emerged, modes of exclusion are breaking down marking new segregated spaces of spatial confrontations shaped by economic neoliberal imperatives (Giroux, 2014). As such disability is not natural nor neutral, but politically determined, forever shifting, resulting in new forms of segregation, territorialized spaces and discrimination. People's identities reflect the territorialized space, time, and context in which those identities were formed; that is, identities change. And as Arendt (1970, p. 76) noted with issues of “race”, equally applicable to “disability” that it, “*is not a fact of life, but an ideology, and the deeds it leads to are not reflex actions, but deliberate acts based on pseudo‐scientific theories*”. Disability is ideologically constructed. Little would it have been known that for some (Ainscow et al., 2006), knowing that UK government policy related to developing inclusion is ambiguous and contradictory, that a further fragmentation of educational provision is taking shape that is pro‐segregation (D*f*E & D*f*HSC, 2022).

At the heart of this conceptual exploration is the major issue of human existence. Certainly, the lives of disabled people have been framed by dominant and dominating paradigms, often with fatal outcomes (Evans, 2004; Kikabhai, 2014; Klee et al., 1988), involving power/knowledge discourses of surveillance, control, regulation, discipline, punishment and exclusion (Kikabhai, 2018). In this modern episteme “*w*e *are segmented from all around in every direction. … in a binary fashion, following the great major dualist oppositions …*” (Deleuze & Guattari, 2008, p. 230). This has been amidst acts of resistance, challenging “*dualisms such as body/mind, normal/abnormal, able/disabled* (symbolic too: 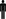/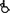) …” (Kikabhai, 2014, p. 149), in opposition to psycho‐hierarchical structures of modernism, “*the impaired body as breaking with repressive and modernist modes of existence constructed by power relations and hierarchically ordered by contrasting dis/abled identities*” (Kikabhai, 2018, pp. 52–53), and offering different possibilities of resistance (symbolic too: 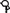/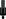). For sure, the history of academic disciplines, can be written in terms of “*violent*” paradigmatic shifts (Lőwith, 1993, p. 1). The geopolitical map has been changing (Newman, 1999), social (class) divisions are deepening, ageism, racism, sexism, antisemitism, homophobia, transphobia, Islamophobia, xenophobia, xeno‐racism, disablism (ableism in the US) are on the rise, inequalities and discriminatory practices have increased and as Fraser (2008) highlights in “*abnormal*” times, amidst global insecurity, growing inequality, displaced and stateless individuals, rising in‐work poverty, food poverty, climate catastrophe, financial crisis, nationalism, rise in national and global territorial disputes, there are now problems of framing justice in a globalizing world. Given the “*all‐subjected principle*” (Fraser, 2008, p. 95) in terms of access to justice, in a post‐Covid‐19 world, it appears that disabled people who are subjected to policies that segregate them are at the same time denied any parity of participation in the formal political process in which to hold the decision‐makers to account. This, almost 4 decades later being a prophetic articulated point made earlier by Williamson (1981). In this sense and in relation to higher education participation; what is its substance and frame of justice that address issues of recognition, redistribution and representation in a globalizing age? Given this context it is important to recognize the relationship between schools and universities, as Slee and Weimar noted:

Schooling has acted as the (credentialing) turnstile for higher education and the skilled and profession‐based workforce. The requirements of the unskilled labour market, together with domestic family service for girls and a segregated system of special [sic] education have colluded with schools to disguise the extent of educational failure. (Slee & Weimar with Tomlinson, 1998, p. 3)

Absolutely, schools continue to have this role, and of course needless to say this is an inaccessible credentialing turnstile for higher education. Arguably, in terms of overcoming injustice, recent examination chaos in the UK, and dismantling institutional and political obstacles, scrutiny also involves an investigation of professional values, those who perpetuate the use of and principally define the “needs” of disabled people including those labeled as having “SEN”. In a recent United Nations concluding observation of the UK on the Convention on the Rights of Persons with Disabilities, with regards to Education (Article 24) reported that the committee is concerned at:

(a) The persistence of a dual education system that segregates children with disabilities, in special schools, including based on parental choice; (b), the increasing number of children with disabilities in segregated education environments; (c) The fact that the education is not equipped to respond to the requirements of high‐quality inclusive education, particularly reports of school authorities refusing to enrol a student with disabilities who is deemed to be “disruptive to other classmates”; (d) The fact that the education and training of teachers in inclusion competences does not reflect the requirements of inclusive education. (UNCRPD, 2017, n.p)

For sure, disabled people whether children or adults should be part of the decision‐making process (participatory parity), to inform and respond to requirements of inclusive education. Far from this, and moreover, the so‐called “helping” and “well‐being” professions have shifted from explicit forms of terror and punishment to more subtle forms of coercive control and self‐regulation (Deleuze & Guattari, 2008; Foucault, 1980). In Fraser's (2008) terms individuals are denied interacting with “others” as peers and suffer from status inequality and distributive injustice. As to the notion of “needs”, Haines and Ruebain (2011, p. 5) noted that “*[t]he emphasis should be on the language of entitlements rather than that of needs*”, and similarly by Morgan, who in the context of higher education, noted:

… the support required by a student is often referred to as “needs”, which is a negative term and suggests that an institution is having to provide extra support to help them succeed. In fact, the support they require is an entitlement, and it is a university's duty and responsibility to provide the support required by a student to enable them to thrive and be successful in their studies. (Morgan, 2013, p. 8)

To be clear, whilst needs‐based provision maybe the “*most redistributive*” they certainly compound “*isolating and stigmatizing*” the so called “*needy*” (Fraser, 1997, p. 50). As noted “needs” reinforce deficit and disadvantage, and taking the pseudo‐medical example of “Emotional and Behavioral Difficulties” induces a clinical mindset “*from which it is difficult to escape*” (Thomas & Loxley, 2001, p. 48). As was pointed out (Kikabhai, 2018), it ought to be borne in mind that throughout history, and across cultures, the actions and practice of labeling (misframing) is determined by judgments made by others including professionals, policy makers, organizations, institutions, researchers, and receptionists. As Fraser (2003, 2008) argued, this is much more than maldistribution or misrecognition but both. In a previous study (Kikabhai, 2014), Val, for example, one of the case study participant's reported that whilst interviewing for a lecturing post:

One of them wasn't quite sure where he was and a member of the reception staff came up to us and said one of your students is lost, in a way that she wouldn't have said with someone who didn't have “learning difficulties” and that's a very day‐to‐day domestic level, and there's a hell of lot of learning that needs to come out, … It will be about confronting their own prejudices and assumptions rather than anything else, and that people with “learning difficulties” aren't any different from anybody else, … (Val quoted by Kikabhai, 2018, p. 191)

What became apparent with the identification of the attitudinal barriers is not only the shift required from higher education but also the assumption that individuals described as having so‐called “learning difficulties” do not attend job interviews; and certainly not, it would seem, for a position as a lecturer. As such, these are complex political social orderings involving misrepresentation, maldistribution and misrecognition (Fraser, 2008, p. 18) and their converse being “*three fundamental dimensions of justice*”.

## 7 CONCLUSION

This paper has focused on how educational systems respond to diversity, inclusion and social justice across territorialized boundaries and how disability is often (intentionally) ignored, silenced and erased. Indeed, as Davis (1995, p. 1) had noted much earlier in terms of the usual repeated triad of “race”, class and gender; disability is a “*missing term*”. This paper specifically drew upon the work of Fraser (1997, 2003, 2008), which has provoked a different set of questions in terms of recognition, redistribution and representation. Related questions are not those that relate to lack, individual deficit or even whether the status of disabled people is better or worse, but those that examine territorial boundaries, surveying territories of knowledge(s) which often shape understanding of equality and social justice from which, arguably, these inequalities emerge. For sure focusing on schools alone is increasingly the wrong place to look. As has been repeatedly shown, education has been used to perpetuate a narrowly defined notion of meritocracy, only to advance the already advantaged. For sure modern higher education is transforming, carving out new territorialized virtual spaces, increasingly subject to neoliberal market and consumerist forces. Indeed, higher education now falls under the Consumer Rights Act 2015. What has been experienced is the discourse of widening participation in relation to disabled people is one of misrecognition, maldistribution, and misrepresentation through an individual deficit, needs‐based, medical, colonial and pathologizing bureaucratic machine. Research too often ignores, silences and erases disability as it intersects with identity characteristics. Epistemic territorialized boundaries of knowledges about disability and its associated discourses, (mis)representations, images, symbols, ideologies cut across educational sectors, and as noted are expressions of power relations. Unashamedly, universities can be found to be engaging in conversations of diversity, inclusion, social justice and more recently decolonization, whilst simultaneously supporting and sponsoring territorialized segregated schooling. Several studies have explored inequalities in higher education, and used social mobility as a measure of success, yet the very system of higher education is situated within a broader social political system. Higher education is part of the problem, a contradiction. Their role in reducing and challenging inequality, only operationalizes social selection rendering social mobility a mirage. Indeed, as is apparent, with reference to disabled people, (one‐generational upward) social mobility, schooling, higher education participation, teacher education, and “special” education programmes carry within them examples of misrecognition, misrepresentation, and maldistribution given their commitment to normalcy, dynamics of a social system, an ideological justification, social selection across territorialized boundaries, perpetuating multiple inequalities, in practice their policy context are laden with ableist/disablist rhetoric, situated within a modern performative regulatory bureaucratic machine, and are far removed from epistemic justice, or to creating accessible and enabling spaces and even further away from a three‐dimensional theory of justice as advanced by Fraser (2008). New territorialized boundaries of segregated spaces have emerged resulting in new forms of segregation and exclusion. Amidst these territorialized and reterritorialized spaces different acts of resistance are challenging dominant paradigms, making transparent the order of things, at times emerging from chance events, from the margins, rhizomatic not tree like, and challenging complex political social orderings.

## Data Availability

All data analyzed during this study are included in this published article.

## ENDNOTES

^1^
Read Oliver (1990), for an understanding of the individual deficit (medical) model of disability.

^2^
Read: Kikabhai (2018). Using various theoretical and conceptual ideas the work argues that “learning difficulties” is a fictious entity, a contested term, not neutral, part of a cultural landscape which is shaped to legitimize processes of exclusion.
